# Liver Enzymes in Early to Mid-pregnancy, Insulin Resistance, and Gestational Diabetes Risk: A Longitudinal Analysis

**DOI:** 10.3389/fendo.2018.00581

**Published:** 2018-10-02

**Authors:** Yeyi Zhu, Monique M. Hedderson, Charles P. Quesenberry, Juanran Feng, Assiamira Ferrara

**Affiliations:** ^1^Women and Children's Health Section, Division of Research, Kaiser Permanente Northern California, Oakland, CA, United States; ^2^Biostatistics Core, Division of Research, Kaiser Permanente Northern California, Oakland, CA, United States

**Keywords:** gestational diabetes, liver enzymes, longitudinal associations, pregnancy, repeated measures

## Abstract

**Background:** Liver enzymes may be implicated in glucose homeostasis; liver enzymes progressively change during pregnancy but longitudinal data during pregnancy in relation to insulin resistance and gestational diabetes (GDM) risk are lacking. We investigated longitudinal associations of γ-glutamyl transferase (GGT) and alanine aminotransferase (ALT) with insulin secretion and resistance markers across early to mid-pregnancy and subsequent GDM risk.

**Methods:** Within the prospective Pregnancy Environment and Lifestyle Study cohort, 117 GDM cases were ascertained and matched to 232 non-GDM controls in a nested case-control study. Fasting blood samples were collected at two clinic visits (CV1, gestational weeks 10–13; CV2, gestational weeks 16–19). Linear mixed model and conditional logistic regression were used, adjusting for major risk factors for GDM.

**Results:** In repeated measure analysis, after adjusting for confounders including body mass index and waist-to-hip ratio, GGT per standard deviation increment was associated with elevated fasting glucose and HOMA-IR (% change = 1.51%, 95% CI 0.56–2.46% and 7.43%, 95% CI 1.76–13.11%, respectively) and decreased adiponectin (% change = −2.86%, 95% CI−5.53 to −0.20%) from CV1 to CV2. At CV1 and CV2, GGT levels comparing the highest versus lowest quartile were associated with 3.01-fold (95% CI 1.32–6.85) and 3.51-fold (95% CI 1.37–8.97) increased risk of GDM, respectively. Progressively increased (<median at CV1, ≥median at CV2) and stably high (≥median at both CV1 and CV2) GGT levels were associated with 3.89- and 2.39-fold increased risk of GDM, compared to stably low levels (<median at both CV1 and CV2), respectively (both *P* < 0.05). Similar but non-significant trends were observed for ALT.

**Conclusion:** Elevated levels of GGT in early and mid-pregnancy, even within the conventional normal range, and its progressive increase from early to mid-pregnancy may be implicated in the pathogenesis of GDM, highlighting its potential to inform early screening or preventive strategies to mitigate subsequent risk of GDM.

## Introduction

Gestational diabetes (GDM), the most common metabolic dysfunction during pregnancy, affects ~15% of pregnant women worldwide (1). The alarming rise in its prevalence over recent decades (2, 3) may be fueling the growing global epidemic of type 2 diabetes (4), becoming a major public health concern. While the underlying etiology remains to be fully understood, β cell dysfunction and thus failure to compensate for insulin resistance induced by pregnancy have been implicated in GDM development (5).

The liver, a major site of insulin action and clearance, plays an important role in maintaining glucose and insulin homeostasis, and thus is recognized as a major target of injury induced by insulin resistance and other metabolic impairments (6). Liver enzymes, specifically γ-glutamyl transferase (GGT, a marker for alcohol-related liver disease and non-alcoholic related liver fat) and alanine aminotransferase (ALT, a marker for hepatocellular damage), even within the normal range, have been linked to a multitude of cardiometabolic diseases including type 2 diabetes (7–9). Nonetheless, data among pregnant women are limited.

Importantly, emerging, yet sparse data indicate that liver enzymes may undergo progressive physiologic changes during pregnancy due to alterations in hormone homeostasis and hemodilution, which concomitantly impact hepatic function (10–12). Thus, a comprehensive understanding of the role of liver enzymes in GDM pathogenesis requires longitudinal investigations throughout pregnancy. Nonetheless, longitudinal and prospective data on liver enzymes throughout early to mid-pregnancy in relation to subsequent risk of GDM are lacking. Further, despite the emerging data that indicate elevated liver enzymes even within the normal range may stimulate insulin resistance among non-pregnant individuals free of diabetes (13), little is known about the patterns of liver enzymes across gestation in relation to insulin secretion and resistance prior to the diagnosis of GDM and subsequent risk of GDM.

Therefore, to address the critical evidence gaps, we aimed to investigate the longitudinal associations of liver enzymes GGT and ALT with markers and indices of insulin secretion and resistance with repeated measures from early to mid-pregnancy and subsequent risk of GDM, in a case-control study nested within a prospective cohort of pregnant women.

## Materials and methods

### Study population and design

The study participants were from the Pregnancy Environment and Lifestyle Study (PETALS), a longitudinal prospective multi-racial/ethnic cohort of pregnant women. The study design and scope have been described in detail elsewhere (14). Briefly, after weekly search of the electronic health records, pregnant women aged 18–45 years, of all races/ethnicities, carrying a singleton, and without recognized pre-existing diseases (i.e., diabetes, cancer, hepatitis C, or liver cirrhosis) were recruited before gestational week 11 at five participating medical centers of Kaiser Permanente Northern California. Questionnaire data and fasting blood specimens were longitudinally collected during early and mid-pregnancy at clinical visit 1 (CV1; gestational weeks 10–13) and CV2 (gestational weeks 16–19), respectively. The study was approved by the human subjects committee of the Kaiser Foundation Research Institute. Informed consent was obtained from all participants.

From October 2013 to June 2016, 1,708 pregnant women were enrolled and delivered a singleton, of which 1,616 (95%) were screened for GDM, serving as the source cohort. To investigate the pathophysiology of GDM, we conducted a nested case-control study within the PETALS cohort, including117 GDM cases and 232 controls individually matched at a ratio of 1:2 (with missing blood samples from 2 out of 234 controls). Matching factors included race/ethnicity (Caucasian, non-Caucasian minorities), age (±5 years), calendar time of enrollment (±3 months), and gestational weeks at blood collection (±3 weeks). Measurements of serum liver enzymes and markers of insulin secretion and resistance were obtained using fasting blood specimens collected at CV1 and CV2, respectively.

### Ascertainment of outcome

In this clinical setting, pregnant women are universally screened for GDM by a 50-g, 1-h glucose challenge test (GCT) around gestational weeks 24–28. Among pregnancies with GCT values above 7.8 mmol/L, a diagnostic 100-g, 3-h oral glucose tolerance test was performed after a 12-h overnight fast and GDM was ascertained according to the Carpenter and Coustan criteria with two or more values meeting or exceeding the following thresholds: fasting glucose 5.3 mmol/L, 1-h 10.0 mmol/L, 2-h 8.6 mmol/L, and 3-h 7.8 mmol/L (15). Serum glucose measurements for diagnosis of GDM were performed using the hexokinase method at the KPNC regional laboratory, which participates in the College of American Pathologists' accreditation and monitoring program (2).

### Measurement of liver enzymes

Fasting blood samples were collected after an 8-12 h overnight fast at CV1 (gestational weeks 10–13) and CV2 (gestational weeks 16–19) and were stored at −80°C until being thawed immediately before assay. All assays were performed at the Lipid and Apolipoprotein Laboratory at the University of Washington (Seattle, WA) without knowledge of GDM status. All measurements were performed in duplicate and results were reported as the mean. Serum concentrations of GGT and ALT were measured on a Roche Modular P Analytics Chemistry auto-analyzer using Roche Diagnostics reagents (Roche Diagenticas Inc., Indianapolis, IN).

### Markers of insulin secretion and resistance

Serum glucose insulin concentrations were measured using a glucose analyzer (YSI 2300 STAT Plus, Yellow Springs, OH) and Millipore radioimmunoassay (St Charles, MO), respectively. The updated homeostatic model assessment for β-cell function (HOMA2-β) and insulin resistance (HOMA2-IR) were calculated by the computer-based models accounting for variations in hepatic and peripheral glucose resistance (16, 17). Adiponectin, as an indicator of insulin resistance (18), was measured by a commercially available radioimmunoassay (Millipore). All the inter- and intra-assay coefficients of variation were <6.2%.

### Covariates

Data on demographic, lifestyle, and clinical factors were obtained from structured questionnaires administered at CV1 and extracted from medical records. Covariates were *a priori* selected: family history of diabetes (yes, no), pre-gestational hypertension (yes, no), alcohol consumption before and/or during pregnancy (≥1 drink/day or not), and pre-pregnancy body mass index (BMI, < 18.5, 18.5–24.9, 25.0–29.9, ≥30.0 kg/m^2^), and waist-to-hip ratio (WHR, ≥0.85 or <0.85) as an indicator of abdominal obesity (19) which has been linked to both elevated liver enzymes and insulin resistance (20). Matching factors age (years), race/ethnicity (non-Hispanic White, African American, Hispanic, Asian/Pacific Islander, other), and gestational week of blood collection (weeks) were also included as covariates to account for residual confounding due to matching ranges and to derive conservative risk estimates. Additional covariates including education, parity, smoking before/after pregnancy, physical activity during pregnancy, diet during pregnancy, prenatal supplement use, and fetal sex were considered but were not retained in the final models failing the inclusion criteria of ≥10% change in the main effect estimates.

### Statistical analysis

Differences in participant characteristics and log-transformed serum GGT and ALT concentrations at the two clinic visits prior to GDM diagnosis between GDM cases and matched controls were assessed by linear mixed models for continuous variables with a random effect for the matched case-control pairs, and by binomial or multinomial logistic regression with generalized estimating equations for binary or multilevel categorical variables. Comparisons of log-transformed GGT or ALT concentrations between clinic visits were obtained by paired *t*-test within cases and controls, respectively.

We first assessed the continuous associations between liver enzymes and markers of insulin secretion and resistance among the entire sample. Specifically, in repeated measures analysis, we assessed the longitudinal associations between time-varying liver enzymes and time-varying markers and indices implicated in glucose and insulin homeostasis (i.e., glucose, insulin, HOMA2-β, HOMA2-IR, and adiponectin) using linear mixed models with subject-specific random intercepts, an auto-regressive covariance structure, and also a random effect for the matched cases-control pairs, adjusting for aforementioned covariates and GDM status. Natural log transformations were performed on aforementioned markers and indices to approximate normal distributions; β coefficients indicated the percent difference in these markers per standard deviation (SD) increase in serum GGT or ALT across CV1 and CV2. Further, stratified analysis was conducted to explore heterogeneity in effects by GDM status among cases and controls, respectively.

Conditional logistic regression models adjusting for covariates were fitted to assess the associations of GGT or ALT at CV1 and CV2 with subsequent risk of GDM, respectively. We analyzed each liver enzyme by categorizing the measurements into quartiles based on the distribution among controls and also by treating each enzyme as a continuous variable standardized by the SD of the measurements among controls. Tests of linear trend were conducted by using the median value for each quartile and fitting it as a continuous variable in the conditional logistic regression models. Further, to investigate the effect of progression and regression of GGT or ALT across early (CV1) to mid-pregnancy (CV2) on subsequent risk of GDM, we assessed the risk estimates associated with joint categories of GGT or ALT levels above or below the respective median at CV1 or CV2 based on distributions among controls. Specifically, stably low was defined as concentrations below median (low) at both CV1 and CV2 (reference group), progression as low at CV1 and high (≥median) at CV2, regression as high at CV1 and low at CV2, and stably high as above median at both visits.

To examine whether insulin resistance could partially explain the associations of GGT or ALT levels in early and mid-pregnancy and their respective changes across early to mid-pregnancy with subsequent risk of GDM, we conducted sensitivity analysis by additionally adjusting for HOMA2-IR as a marker of insulin resistance. Further, to test the robustness of our results against the potential impact of pathophysiologically elevated liver enzymes, we conducted sensitivity analyses by excluding women with GGT or ALT levels above the normal range established among non-pregnant women [GGT >33 U/L (21)] and ALT >19 U/L (22), due to lack of normal references tailored to pregnant women. We also assessed the potential effect modification by status of overall overweight/obesity (BMI ≥ 25 kg/m^2^), abdominal obesity (WHR ≥ 0.85), and race/ethnicity (non-Hispanic White, African American, Hispanic, Asian/Pacific Islander, other). Interaction was examined by likelihood ratio test. Moreover, we excluded women with hepatitis C at the enrollment given that GGT and ALT are biomarkers more specific to chronic hepatitis C compared to hepatitis A or B (23, 24). Nonetheless, we also conducted sensitivity analysis by further excluding women with self-report or physician diagnosis of hepatitis A or B (*n* = 3). All analyses were conducted using SAS version 9.4 (SAS Institute, Cary, NC).

## Results

**Figure 1:**
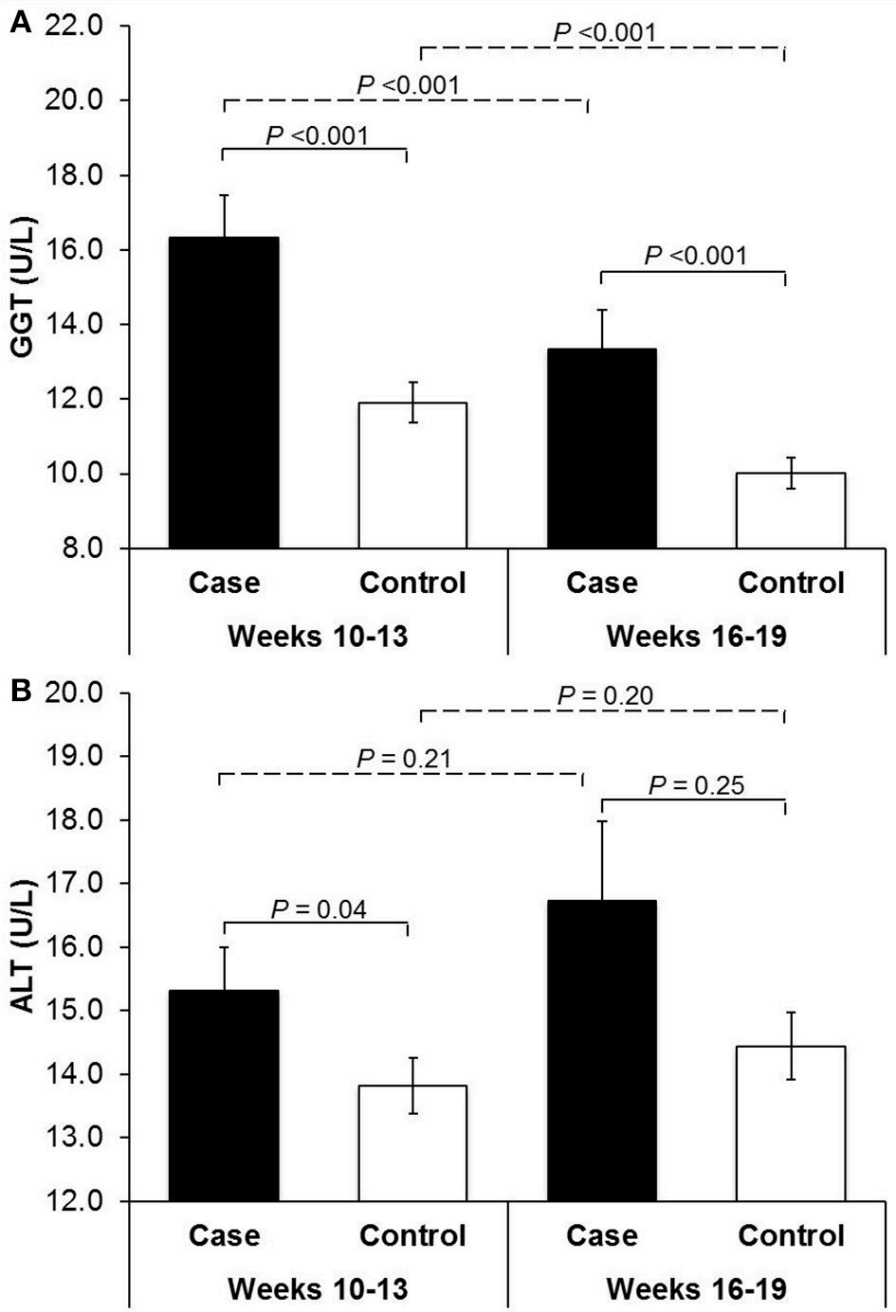
Serum mean concentrations of **(A)** γ-glutamyl transferase and **(B)** alanine aminotransferase among women with gestational diabetes and their matched controls at gestational weeks 10–13 and 16–19. ALT, alanine aminotransferase; GGT, γ-glutamyl transferase. *P* values for case-control comparisons of the log-transformed GGT or ALT levels were obtained by linear mixed-effect linear regression models with a random effect for the matched case-control pairs at gestational weeks 10–13 and 16–19, respectively. *P* values for between-visit comparisons were obtained by paired *t*-test within cases and controls, respectively.

Compared to non-GDM controls, women with GDM were more likely to have pre-gestational hypertension and a higher prepregnancy BMI but less likely to have alcohol use during 3 months before pregnancy (Table 1). Compared to non-GDM controls, GDM cases had significantly higher levels of GGT at both CV1 (gestational weeks 10–13) and CV2 (weeks 16–19) and higher levels of ALT at CV1. Across CV1 and CV2, GGT concentrations tended to decrease regardless of GDM status, whereas ALT concentrations slightly increased although to a non-statistically significant extent (Figure 1).

**Table 1:** Participant characteristics among gestational diabetes cases and non-gestational diabetes controls: a nested case-control study within the PETALS prospective pregnancy cohort, 2013-2016.

	**GDM case**	**Non-GDM control**	***P*-value^a^**
	**(*n* = 117)**	**(*n* = 232)**	
Age, *n (%)*, years			0.59
18-24	5 (4.3)	16 (6.9)	
25-29	24 (20.5)	51 (22.0)	
30-34	56 (47.9)	114 (49.1)	
≥35	32 (27.4)	51 (22.0)	
Race/Ethnicity, *n (%)*			0.76
Non-Hispanic White	23 (19.7)	49 (21.1)	
African American	7 (6.0)	22 (9.5)	
Asian	45 (38.5)	74 (31.9)	
Hispanic	42 (35.9)	87 (37.5)	
Education, *n (%)*			0.77
High school or less	12 (10.3)	23 (9.9)	
Some college	47 (40.2)	91 (39.2)	
College graduate or above	57 (48.7)	118 (50.9)	
Missing	1 (0.9)	0 (0.0)	
Parity, *n (%)*			0.88
0	48 (41.0)	93 (40.1)	
1	40 (34.2)	84 (36.2)	
≥2	29 (24.8)	52 (22.4)	
Missing	0 (0.0)	3 (1.3)	
Pre-pregnancy body mass index, *n (%), kg/m^2^*			<0.0001
< 18.5	1 (0.9)	6 (2.6)	
18.5-24.9	20 (17.1)	100 (43.1)	
25.0-29.9	36 (30.8)	56 (24.1)	
≥30.0	60 (51.3)	70 (30.2)	
Family history of diabetes, *n (%)*	36 (30.8)	53 (22.8)	0.13
Pre-gestational hypertension, *n (%)*	11 (9.4)	10 (4.3)	0.05
Smoking during 1 mo preceding pregnancy, *n (%)*	11 (9.4)	16 (6.9)	0.63
Smoking in early pregnancy, *n (%)*	1 (0.9)	1 (0.4)	0.39
Alcohol use during 3 mos preceding pregnancy, *n (%)*	48 (41.0)	131 (56.5)	0.004
Alcohol use in early pregnancy, *n (%)*	13 (11.1)	44 (19.0)	0.07

*Data are presented as n (%) for categorical variables*.

a *Obtained by binomial/multinomial logistic regression with generalized estimating equations for binary/multilevel categorical variables, accounting for matched case-control pairs*.

Among the entire sample, in repeated analysis, increased GGT but not ALT concentrations over early to mid-pregnancy were associated with markers of insulin resistance (Table 2). Overall, per SD (9.2 U/L) increase of GGT from early to mid-pregnancy was associated with 1.51% increase in fasting glucose (*P* = 0.002), 8.36% increase in insulin (*P* = 0.009), 7.43% increase in HOMA2-IR (*P* = 0.010), and 2.86% decrease in adiponectin (*P* = 0.035), after adjusting for aforementioned covariates and GDM status. No associations were observed for GGT in relation to HOMA2-β. When stratified by GDM status, significant associations were only evident among GDM cases but not controls. Similar trends were observed in longitudinal associations between ALT and these markers; however, risk estimates were not statistically significant.

**Table 2:** Adjusted percent difference in glucose and insulin homeostasis markers per one standard deviation increase in serum γ-glutamyl transferase or alanine aminotransferase concentrations from early to mid-pregnancy^a^.

**Liver enzymes**	**Glucose and insulin homeostasis markers**	**All (*****n*** = **349)**		**GDM cases (*****n*** = **117)**		**Non-GDM controls (*****n*** = **232)**	
		**β (95% CI)**	***P***	**β (95% CI)**	***P***	**β (95% CI)**	***P***
**GGT (U/L)**^b^	Glucose (mg/dL)	1.51 (0.56, 2.46)	0.002	2.23 (0.48, 3.99)	0.013	0.56 (−0.62, 1.74)	0.349
	Insulin (μU/mL)	8.36 (2.11, 14.61)	0.009	11.35 (2.21, 20.49)	0.015	5.56 (−3.51, 14.64)	0.228
	HOMA2-β	2.17 (−1.34, 5.68)	0.224	2.80 (−2.00, 7.59)	0.250	2.11 (−3.11, 7.321)	0.427
	HOMA2-IR	7.43 (1.76, 13.11)	0.010	9.84 (2.05, 17.62)	0.014	5.03 (−3.43, 13.48)	0.243
	Adiponectin (ng/mL)	−2.86 (−5.53, −0.20)	0.035	−3.90 (−7.81, −0.01)	0.048	−0.48 (−2.74, 1.77)	0.673
**ALT (U/L)**^c^	Glucose (mg/dL)	−0.02 (−0.86, 0.82)	0.958	−0.20 (−1.81, 1.41)	0.806	0.32 (−0.63, 1.27)	0.508
	Insulin (μU/mL)	1.82 (−3.58, 7.23)	0.508	1.72 (−7.10, 10.53)	0.701	1.58 (−5.50, 8.67)	0.660
	HOMA2-β	1.21 (−1.68, 4.10)	0.411	2.31 (−1.80, 6.42)	0.268	0.16 (−3.85, 4.16)	0.938
	HOMA2-IR	2.23 (−2.62, 7.08)	0.367	3.01 (−4.39, 10.42)	0.422	1.26 (−5.30, 7.83)	0.705
	Adiponectin (ng/mL)	−0.56 (−2.16, 1.04)	0.491	−0.52 (−2.84, 1.79)	0.655	−0.48 (−2.74, 1.77)	0.673

*ALT, alanine aminotransferase; GGT, γ-glutamyl transferase; HOMA2-β, updated homeostatic model assessment of β-cell function; HOMA2-IR, updated homeostasis model assessment of insulin resistance*.

a *Repeated measures analysis was adjusted for age, race/ethnicity, gestational week at blood collection, family history of diabetes, pre-gestational hypertension, alcohol use before and/or during pregnancy, pre-pregnancy body mass index, waist-to-hip ratio, and gestational diabetes (not in the stratified analysis)*.

b *Standard deviation of GGT: 9.2 U/L*.

c *Standard deviation of ALT: 8.4U/L*.

We further examined associations of liver enzymes in early to mid-pregnancy with subsequent risk of GDM, respectively (Table 3). At CV1 (gestational weeks 10–13) and CV2 (weeks 16–19), GGT in the highest versus the lowest quartile was associated with a 3.01- and 3.51-fold increased risk of GDM after adjusting for covariates, respectively (both *P*-for-trend < 0.05, model 2). A linear relationship was observed when liver enzymes were parameterized continuously, with a 1.43- and 1.45-fold increased risk of GDM per SD increase in GGT levels at gestational weeks 10–13 and 16–19, respectively (model 2). On the other hand, at weeks 10-13, ALT comparing the highest vs. lowest quartile was significantly associated with a 2.05-fold [95% confidence interval (CI) 1.06, 3.99; *P*-for-trend = 0.035; model 2]; however, the significant association did not persist after adjusting for covariates. In the sensitivity analysis with additional adjustment for HOMA2-IR as a marker of insulin resistance (model 3), the results were slightly attenuated but remained significant.

**Table 3:** Crude and adjusted odds ratio (95% CI) of gestational diabetes associated with γ-glutamyl transferase or alanine aminotransferase during early-to-mid pregnancy.

	**Crude model**	**Model 1^a^**	**Model 2^b^**	**Model 3^c^**
**Gestational weeks 10–13**				
**GGT, U/L**				
Q1: 3–7^d^	1	1	1	1
Q2: 8–10	1.51 (0.75, 3.05)	1.76 (0.83, 3.70)	2.05 (0.88, 4.78)	1.97 (0.80, 4.85)
Q3: 11–14	2.22 (1.07, 4.61)	2.31 (1.06, 5.04)	2.82 (1.17, 6.76)	2.75 (1.10, 6.88)
Q4: 15–81	3.78 (1.93, 7.39)	3.81 (1.84, 7.91)	3.01 (1.32, 6.85)	2.93 (1.26, 6.80)
*P*-for-trend	<0.001	<0.001	0.011	0.016
Per SD increment	1.58 (1.25, 1.99)	1.58 (1.23, 2.03)	1.43 (1.08, 1.90)	1.39 (1.03, 1.87)
**ALT, U/L**				
Q1: 5–9^d^	1	1	1	1
Q2: 10–12	1.35 (0.71, 2.59)	1.47 (0.73, 2.94)	1.38 (0.63, 3.02)	1.28 (0.57, 2.91)
Q3: 13–16	1.34 (0.67, 2.65)	1.65 (0.79, 3.44)	1.08 (0.47, 2.49)	1.03 (0.43, 2.47)
Q4: 17–54	2.05 (1.06, 3.99)	2.44 (1.18, 5.02)	1.73 (0.75, 3.95)	1.56 (0.66, 3.70)
*P*-for-trend	0.035	0.015	0.247	0.367
Per SD increment	1.31 (1.04, 1.64)	1.34 (1.04, 1.71)	1.17 (0.87, 1.55)	1.11 (0.82, 1.49)
**Gestational weeks 16–19**				
**GGT, U/L**				
Q1: 3–6^d^	1	1	1	1
Q2: 7–8	2.86 (1.36, 6.02)	3.09 (1.30, 7.36)	3.03 (1.23, 7.46)	3.01 (1.21, 7.29)
Q3: 9–12	3.10 (1.35, 7.11)	3.27 (1.42, 7.55)	3.36 (1.30, 8.65)	3.30 (1.24, 8.14)
Q4: 13–47	4.02 (1.87, 8.65)	3.92 (1.73, 8.86)	3.51 (1.37, 8.97)	3.41 (1.31, 9.02)
*P*-for-trend	0.001	0.002	0.024	0.030
Per SD increment	1.55 (1.20, 1.99)	1.56 (1.19, 2.06)	1.45 (1.06, 1.98)	1.41 (1.04, 1.93)
**ALT, U/L**				
Q1: 4–10^d^	1	1	1	1
Q2: 11–12	0.74 (0.31, 1.79)	0.73 (0.29, 1.85)	0.68 (0.24, 1.93)	0.67 (0.23, 1.93)
Q3: 13–16	0.78 (0.40, 1.52)	0.89 (0.44, 1.80)	0.55 (0.24, 1.27)	0.53 (0.21, 1.26)
Q4: 17–71	1.35 (0.72, 2.53)	1.35 (0.68, 2.70)	1.12 (0.51, 2.42)	1.11 (0.48, 2.46)
*P*-for-trend	0.31	0.305	0.628	0.652
Per SD increment	1.17 (0.93, 1.48)	1.18 (0.91, 1.53)	1.15 (0.87, 1.52)	1.10 (0.83, 1.51)

*ALT, alanine aminotransferase; GGT, γ-glutamyl transferase; Q, quartile; SD, standard deviation*.

a *Adjusted for age (years), race/ethnicity (White, African American, Asian/other, Hispanic), gestational week at blood collection (weeks), family history of diabetes (yes, no), pre-gestational hypertension (yes, no), and alcohol use before and/or during pregnancy (≥1 drink/day or not)*.

b *Adjusted for covariates in Model 1, pre-pregnancy body mass index (< 18.5, 18.5–24.9, 25.0–29.9, ≥30.0 kg/m^2^), and waist-to-hip ratio (≥0.85 or not)*.

c *Sensitivity analysis: Adjusted for covariates in Model 2 and updated homeostasis model assessment of insulin resistance*.

d *Quartiles are classified based on distributions of biomarkers among non-gestational diabetes controls*.

**Figure 2:**
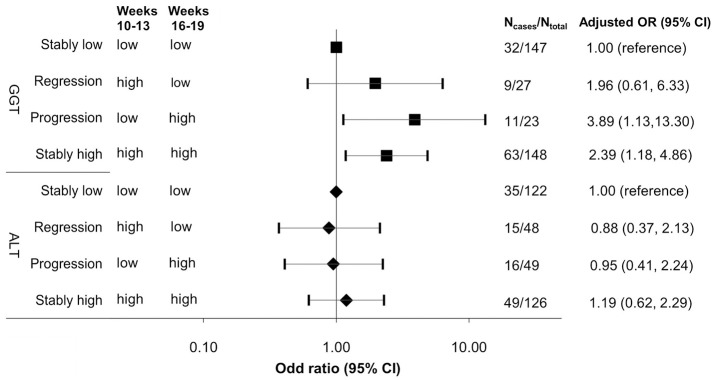
Adjusted odds ratio (95% CI) of GDM risk associated with progression and regression of γ-glutamyl transferase and alanine aminotransferase from early to mid-pregnancy, ALT, alanine aminotransferase; GGT, γ-glutamyl transferase; stably low, levels <median at both visits; regression: levels ≥median at visit 1 (12 U/L for ALT, 10 U/L for GGT) and <median at visit 2 (12 U/L for ALT, 8 U/L for GGT); progression: levels <median at visit 1 and ≥median at visit 2; stably high: levels ≥median at both visits; early pregnancy: clinic visit 1 (gestational weeks 10–13); mid-pregnancy: clinic visit 2 (gestational weeks 16–19). Risk estimates were adjusted for age (years), race/ethnicity (White, African American, Asian/other, Hispanic), difference in gestational week at blood collection between clinic visits 1 and 2 (weeks), family history of diabetes (yes, no), pre-gestational hypertension (yes, no), alcohol use before and/or during pregnancy (≥1 drink/day or not), pre-pregnancy body mass index (<18.5, 18.5–24.9, 25.0–29.9, ≥30.0 kg/m^2^), and waist-to-hip ratio (≥0.85 or not).

Across CV1 and CV2 from early to mid-pregnancy, compared to stably low levels (<median 10 U/L at CV1 and <median 8 U/L at CV2) of GGT, progressively increased levels (<median at CV1 and ≥median CV2) and stably high levels of GGT (≥median at both visits) were associated a 3.89-fold (95% CI 1.13, 13.30) and 2.39-fold (95% CI 1.18, 4.86) increased risk of GDM, whereas progressively regressed (≥median at CV1 and <median CV2) GTT levels was not associated with subsequent risk of GDM (Figure 2). No significant associations were observed for changes in ALT levels across CV1 and CV2. In sensitivity analyses excluding women with elevated liver enzymes based on the normal range established among non-pregnant women (*n* = 13 and 6 for GGT >33 U/L; *n* = 51 and 57 for ALT >19 U/L at CV1 and CV2, respectively), results did not appreciably change (data not shown). Likewise, the results were robust against additional adjustment for HOMA2-IR.

There was significant interaction between GGT and abdominal obesity; the GGT-GDM association was only significant among women with high GGT concentrations (≥median 10 U/L at CV1 or 8 U/L at CV2) and WHR ≥0.85 (*P*-for-interaction = 0.001 and 0.025 at CV1 and CV2, respectively) (Table S1). No significant effect modification was observed for the association between GGT and GDM risk by race/ethnicity (*P*-for-interaction = 0.21). Despite a non-significant association between ALT and GDM risk, combination of high ALT concentrations and overall overweight/obesity or abdominal obesity illustrated a synergistic effect. Compared to women with low ALT (<median 12 U/L at either CV1 or CV2) and BMI <25 kg/m^2^, women with high ALT levels (≥median 12 U/L) and BMI ≥25 kg/m^2^ had a 4.20- and 3.29-fold increased risk of GDM at CV1 and CV2, respectively. Further, in the sensitivity analysis further excluding women with recognized hepatitis A or B (*n* = 3), the results remained similar.

## Discussion

In this case-control study nested within the prospective PETALS cohort, elevated GGT levels as early as the first trimester, even within the conventional normal range established of non-pregnant individuals, were significantly associated with markers of insulin resistance and increased risk of subsequent GDM but not markers of insulin secretion. Further, despite an overall decreasing trend of GGT over early to mid-pregnancy, progressively increased GGT levels from early to mid-pregnancy were associated with almost 4-fold increased risk of GDM.

We are the first study to our knowledge to longitudinally and prospectively examine liver enzymes from early to mid-pregnancy in relation to markers of insulin secretion and resistance and subsequent risk of GDM. Previous studies were largely cross-sectional and based on retrospective data with single measurements coinciding with the time of GDM diagnosis (25–28), precluding conclusions regarding the temporal sequence. Our findings are consistent with data from one study which linked pregravid GGT but not ALT measured on average 7 years preceding the index pregnancy to increased risk of subsequent GDM (29). In contrast, two previous studies reported significant and positive associations between ALT in the first trimester and subsequent GDM risk, although data were not adjusted for important confounders such as WHR as an indicator of abdominal obesity (30, 31), which has been linked to both elevated liver enzymes and insulin resistance (20). Notably, we also observed significant crude associations between ALT at gestational weeks 10–13 and subsequent GDM risk, whereas significant associations did not persist after additionally adjusting for covariates including WHR. Indeed, in our study the association between GGT and GDM appeared to be moderated by abdominal obesity, and in the stratified analysis, only present among women with WHR ≥0.85.

Importantly, our longitudinal data illustrated notable physiologic changes in liver enzymes during pregnancy, particularly a decreasing trend of GGT in contrast to a slightly upward but non-significant trend of ALT from early to mid-pregnancy. This observation was consistent with previous data among 103 healthy pregnant women, likely in response to increases in sex steroid levels and alterations in free fatty-acid metabolism and consequent alterations in hepatic function induced by pregnancy (10–12). Our sensitivity analysis restricted to women with GGT or ALT levels within the normal range established of non-pregnant women found similar results. Taken together, these data call for evaluation of the normal range for liver enzymes during pregnancy, given that high GGT levels even within the conventional non-pregnant normal range were associated with significantly increased risk of GDM. Further, despite the overall decreasing trend of GGT, an increase in GGT from early to mid-pregnancy may prompt further evaluation with respect to subsequent GDM risk. Notably, GDM is conventionally screened for and diagnosed at gestational weeks 24–28, leaving little time for effective interventions or treatment. In this regard, identification of pre-diagnostic markers and its progressive trends for subsequent GDM is warranted (32), which may be utilized to inform early screening or preventive strategies.

Although the exact pathophysiological pathways underlying GDM development remain to be elucidated, our longitudinal data on repeated measures of GGT and ALT from early to mid-pregnancy in relation to markers and indices of insulin secretion and resistance may provide mechanistic insight. In line with our finding that GGT is associated with elevated insulin resistance, animal data demonstrated that hepatic GGT overexpression may induce insulin resistance (33). Concomitantly, epidemiological data among non-pregnant individuals have linked elevated serum GGT to increased insulin resistance and intrahepatic lipids (13, 34) but not β-cell function (34). Moreover, in contrast to ALT, which is predominantly localized in hepatocytes and thus a specific marker for liver injury, GGT is a ubiquitous epithelial enzyme involved in extracellular catabolism of antioxidant glutathione and thus a marker of oxidative stress (35, 36), which in turn may induce insulin resistance (37). Thus, different downstream pathways and cellular processes may partially explain the stronger association of GGT versus ALT with GDM risk. Likewise, outside of pregnancy, synthesized results in a meta-analysis of 10 prospective cohorts with measurements of both GGT and ALT indicate that GGT is a more sensitive marker for incident diabetes (7). Notably, in our sensitivity analysis with additional adjustment for HOMA2-IR as a marker of insulin resistance, the results were slightly attenuated but remained robust, suggesting that insulin resistance may not fully explain the positive association between GGT levels and risk of GDM. Future investigations on other mechanistic pathways may be warranted.

Our study has several notable strengths. The prospective study design is vital to ascertaining the temporal sequence of liver enzymes during early to mid-pregnancy in relation to subsequent GDM risk. Furthermore, longitudinal measurements of markers and indices involved in glucose and insulin homeostasis were also available in the present study, providing a unique opportunity to gain mechanistic insight into the role of liver enzymes in GDM development. We also obtained detailed data on conventional risk factors for GDM including demographic, medical, and lifestyle factors (including physical activity and diet) to minimize potential residual confounding. Finally, we had simultaneous measurements of GGT and ALT. Direct comparison of risk estimates associated with GGT and ALT in the same study setting may shed light onto the existing debate regarding the superior predictive ability of these liver enzymes in hyperglycemic status (7).

Some potential limitations of our study merit discussion. First, we did not have direct assessment of visceral fat to account for its possible residual confounding. Nonetheless, we used WHR in early pregnancy as a proxy, which has been demonstrated as a simple and reliable surrogate measure for intra-abdominal or visceral fat (38). Second, we did not have direct measurement of glucose and insulin homeostasis via the euglycemic clamp technique or insulin suppression test; however, it is experimentally intensive and impractical at large-scale epidemiological investigations. Thus, we utilized the updated computerized models of HOMA2-β and HOMA2-IR, which has been demonstrated reliable and valid to assess longitudinal changes in β-cell function and insulin resistance (17). Further, we conducted sensitivity analyses restricted to women within the normal range of GGT or ALT established among non-pregnant women (21, 22), due to the lack of tailored normal ranges for pregnant women. Indeed, our unique data demonstrated the progressive gestational changes in liver enzymes (particularly GGT), highlighting the importance of further evaluation of physiological normal ranges of these liver enzymes during pregnancy.

In summary, elevated GGT levels as early as gestational weeks 10–13 were significantly associated with markers of insulin resistance and increased risk of subsequent GDM, suggesting that incipient perturbations in glucose and insulin homeostasis are already underway prior to conventional time for GDM screening and diagnosis. Further, from early to mid-pregnancy, despite an overall decreasing trend of GGT, progressively increased levels of GGT elevated subsequent risk of GDM. Our findings suggest the pathophysiological role of GGT as early as the first trimester in GDM development, highlighting its potential to inform early screening or preventive strategies to mitigate subsequent risk of GDM.

## Author contributions

YZ and AF conceived the study concept. YZ performed the statistical analysis and drafted the original manuscript. MH contributed to data interpretation and revised the manuscript. CQ contributed to statistical analysis and data interpretation. JF contributed to data management and analysis. All authors contributed to the interpretation of data discussed in the manuscript, revised the manuscript and approved its final version to be published. YZ and AF are the guarantors of this work and, as such, had full access to all the data in the study and take responsibility for the integrity of the data and the accuracy of the data analysis.

### Conflict of interest statement

The authors declare that the research was conducted in the absence of any commercial or financial relationships that could be construed as a potential conflict of interest. The handling editor declared a past co-authorship with one of the authors YZ.
