# Supplementary material for: Liver Enzymes in Early to Mid-pregnancy, Insulin Resistance, and Gestational Diabetes Risk: A Longitudinal Analysis
Source: Front Endocrinol (Lausanne). 2018 Oct 2;9:581. doi: 10.3389/fendo.2018.00581 (PMC6176077; doi:10.3389/fendo.2018.00581)
Supplement: Supplementary file 1 [file Table_1.DOCX]

Supplementary Material

**Liver enzymes in early to mid-pregnancy, insulin resistance, and gestational diabetes risk: A longitudinal analysis**

**Yeyi Zhu^*^, Monique M. Hedderson, Charles P. Quesenberry, Juanran Feng, Assiamira Ferrara**

*** Correspondence:** Yeyi Zhu: [yeyi.zhu@kp.org](mailto:yeyi.zhu@kp.org)

# Supplementary Table

| **Table S1: Adjusted odds ratio (95% CI) of GDM risk associated with joint effects of liver enzymes and overall overweight/obesity or abdominal obesity**^a^ | | |
| --- | --- | --- |
|  | **Gestational weeks 10-13** | **Gestational weeks 16-19** |
| **Liver enzymes and overweight/obesity** |  |  |
| **GGT (U/L) × BMI (kg/m^2^)** |  |  |
| Low GGT (<median^b^) + BMI <25 | 1 | 1 |
| High GGT (≥median) + BMI <25 | 1.90 (0.69, 5.26) | 0.71 (0.21, 2.34) |
| Low GGT (<median) + BMI ≥25 | **3.80 (1.54, 9.38)** | 1.99 (0.77, 5.18) |
| High GGT (≥median) + BMI ≥25 | **7.58 (2.98, 19.2)** | **5.12 (2.06, 12.7)** |
| *P*-for-interaction^c^ | 0.052 | **0.007** |
| **ALT (U/L) × BMI (kg/m^2^)** |  |  |
| Low ALT (<median^b^) + BMI <25 | 1 | 1 |
| High ALT (≥median) + BMI <25 | 0.82 (0.29, 2.32) | 0.67 (0.21, 2.17) |
| Low ALT (<median) + BMI ≥25 | **3.32 (1.31, 8.39)** | **3.26 (1.20, 8.84)** |
| High ALT (≥median) + BMI ≥25 | **4.20 (1.72, 10.3)** | **3.29 (1.25, 8.65)** |
| *P*-for-interaction^c^ | 0.232 | 0.068 |
| **Liver enzymes and abdominal obesity** |  |  |
| **GGT (U/L) × WHR** |  |  |
| Low GGT (<median^b^) + WHR (<0.85) | 1 | 1 |
| High GGT (≥median) + WHR (<0.85) | 1.10 (0.36, 3.37) | 0.98 (0.30, 3.18) |
| Low GGT (<median) + WHR (≥0.85) | 2.06 (0.83, 5.15) | 2.05 (0.76, 5.53) |
| High GGT (≥median) + WHR (≥0.85) | **5.02 (1.97, 12.8)** | **4.80 (1.71, 13.5)** |
| *P*-for-interaction^c^ | **0.001** | **0.025** |
| **ALT (U/L) × WHR** |  |  |
| Low ALT (<median^b^) + WHR (<0.85) | 1 | 1 |
| High ALT (≥median) + WHR (<0.85) | 1.41 (0.50, 3.92) | 0.69 (0.22, 2.17) |
| Low ALT (<median) + WHR (≥0.85) | **3.46 (1.36, 8.81)** | 2.47 (0.98, 6.25) |
| High ALT (≥median) + WHR (≥0.85) | **3.54 (1.51, 8.26)** | **2.52 (1.01, 6.28)** |
| *P*-for-interaction^c^ | 0.484 | 0.874 |
| ALT, alanine aminotransferase; BMI, pre-pregnancy body mass index (kg/m^2^); GGT, γ-glutamyl transferase; WHR, waist-to-hip ratio.  ^a^Adjusted for age, race/ethnicity, gestational week at blood collection, family history of diabetes, pre-gestational hypertension, alcohol use before and/or during pregnancy, pre-pregnancy body mass index (not in the models for liver enzymes and overweight/obesity), and waist-to-hip ratio (not in the models for liver enzymes and abdominal obesity).  ^b^Median for GGT was 10 U/L at CV1 and 8 U/L at CV2; median for ALT was 12 U/L at both CV1 and CV2.  ^c^Obtained by likelihood ratio test. | | |
